# Social status mediates the propagation of unfairness

**DOI:** 10.3389/fpsyg.2024.1253831

**Published:** 2024-09-09

**Authors:** Hyeran Kang, JuYoung Kim, Daeeun Kim, Hackjin Kim

**Affiliations:** ^1^Laboratory of Social and Decision Neuroscience, School of Psychology, Korea University, Seoul, Republic of Korea; ^2^Department of Psychology, Korea Army Academy at Yeongcheon, Gyeongsangbuk-do, Republic of Korea

**Keywords:** pay-it-forward reciprocity, fairness, hierarchy, dictator game, economic decision-making, sense of power, self-enhancement

## Abstract

Fairness constitutes a cornerstone of social norms, emphasizing equal treatment and equitable distribution in interpersonal relationships. Unfair treatment often leads to direct responses and can spread to others through a phenomenon known as pay-it-forward (PIF) reciprocity. This study examined how unfairness spreads in interactions with new partners who have higher, equal, or lower status than the participants. In the present study, participants (*N* = 47, all Korean) were given either fair or unfair treatment in the first round of a dictator game. They then allocated monetary resources among partners positioned at various hierarchical levels in the second round. Our main goal was to determine if the severity of inequity inflicted on new partners was influenced by their hierarchical status. The results revealed an inclination among participants to act more generously towards partners of higher ranking despite prior instances of unfair treatment, whereas a tendency for harsher treatment was directed towards those with lower ranking. The interaction between the fairness in the first round (DG1) and the hierarchical status of the partner in the second round (DG2) was significant, indicating that the effect of previous fairness on decision-making differed depending on the ranking of the new partners. This study, therefore, validates the presence of unfairness PIF reciprocity within hierarchical contexts.

## 1 Introduction

In Korea, the term “Taeoom,” meaning “burn to ashes,” specifically refers to the physical and psychological bullying of newly employed nurses by senior nurses, a phenomenon that is prominent in South Korea and represents a major unresolved issue (Hutchinson et al., 2009; Seo et al., 2012; Kim and Bae, 2020). This includes actions such as hostile comments, physical violence, persistent criticism, social exclusion, and excessive task allocation (Seo et al., 2012; Oh et al., 2016). The impact of such bullying extends beyond individual suffering, causing depression, suicidal thoughts, post-traumatic stress disorder, and reduced job satisfaction, which ultimately affects the entire organization (Yeun and Kwon, 2007; Kang and Yun, 2016; Kim and Bae, 2020).

This issue is not unique to South Korea; similar phenomena are observed globally (Oh et al., 2016). In Western contexts, the phenomenon mirrors “horizontal hostility” (Farrell, 2001; Bartholomew, 2006) or “lateral violence” (Griffin, 2004; Sheridan-Leos, 2008), illustrating behaviors intended to harm, undermine, and belittle a target who is working at the same professional level (Sanner-Stiehr and Ward-Smith, 2017) or peers (Farrell, 2001), often referred to as “nurses eat their young” (Bartholomew, 2006). Despite differing terminologies, both Taeoom and horizontal hostility highlight the power imbalances between senior and newly employed individuals, making it difficult for the latter to defend themselves effectively (Johnson and Rea, 2009). This cycle of maltreatment continues as victims become bullies when they gain more power, creating a chain of downward unfairness (Cleary et al., 2010; Walrafen et al., 2012; Choeng and Lee, 2016; Kang and Yun, 2016).

Spreading experiences of fairness or unfairness to uninvolved individuals has been revealed in numerous studies (Gray et al., 2014; Hu et al., 2015; Wu et al., 2015; Horita et al., 2016; Strang et al., 2016; Zheng et al., 2017; Hu et al., 2018), where such phenomenon is referred to as pay-it-forward (PIF) reciprocity or generalized reciprocity (Nowak and Sigmund, 2005; Pfeiffer et al., 2005). PIF reciprocity originally describes the spread of benevolence, where one’s act of kindness can lead to acts of goodwill by the recipient and forward in a chain (Brandt and Sigmund, 2004; Desteno et al., 2010; Chang et al., 2012). This behavior supports the maintenance of social norms and a cooperative societies from an evolutionary perspective (Nowak and Sigmund, 2005). This social value begins to develop around the age of four, encouraging humans to extend their cooperative behaviors to new social interactions from an early stage of development (Leimgruber et al., 2014; Beeler-Duden and Vaish, 2020). Furthermore, the principle of pay-it-forward reciprocity extends beyond physical interactions and is notably observed in online environments, where users willingly share valuable information for the benefit of the collective (Suri and Watts, 2011; Yang et al., 2020).

Recent studies have expanded this concept to include fairness-related behaviors in both positive and negative contexts. Participants who received unfair treatment behaved more selfishly to an innocent third person, whereas those who experienced fair treatment in previous interactions exhibited more generous behaviors toward unrelated individuals (Wu et al., 2015; Strang et al., 2016; Hu et al., 2018). Specifically, a fair split of money or labor (i.e., good task: rating humorous stimuli vs. bad task: circling voxels in dense foreign text) led to fair treatment to others, while greedy divisions resulted in unfair treatment to others (Gray et al., 2014). However, the study by Capraro and Marcelletti (2014) challenges this view by demonstrating that good actions do not necessarily inspire subsequent good actions in others. Their findings suggest that the spread of altruistic behavior may not be as robust, adding complexity to our understanding of generalized reciprocity (Capraro and Marcelletti, 2014). Moreover, the extent of spreading unequal division was more significant than that of fair splits in the dictator games, where a single trial consisted of two games, and participants alternated between roles as a recipient and a dictator (Hu et al., 2018). Therefore, principles such as “You scratch my back and I will scratch someone else’s” (Binmore, 1994) and “You pinch my finger and I will pinch someone else’s” highlight the transmission of socially undesirable behaviors like unfairness within human society.

Social hierarchy is another factor that significantly influences selfish motivations (Hoffman et al., 1994), immoral behaviors (Lammers et al., 2010), and the acceptance of unfair offers from high-status partners (Blue et al., 2016). If the proposer’s hierarchy is attained through reasonable standard such as scoring high on a quiz rather than random assignment, they are more likely to exhibit self-centered behaviors in ultimatum and dictator games (Hoffman et al., 1994). The impact of social status on unfairness perception has been demonstrated in prior research (Albrecht et al., 2013; Hu et al., 2015). Individuals endowed with higher status were more likely to reject unfair offers than when endowed with low status (Hu et al., 2015), a phenomenon described as the ‘entitlement effect’ (Hoffman et al., 1994; Ball et al., 2001). This aligns with the observation that higher-ranked individuals tend to express anger more freely and directly when faced with negative events, while lower-ranked individuals are more reluctant to do so due to the anticipated negative social feedback (Petkanopoulou et al., 2019; Van Kleef and Lange, 2020). The hierarchy of both the individual and their partner plays a crucial role in decision-making processes, elucidating why individuals often conform to the opinions and beliefs of higher-ranking individuals (Galinsky et al., 2008; Hays and Goldstein, 2015; Qi et al., 2018). This conformity leads to changes in prior beliefs to align with those of high-ranking partners, particularly in public conditions where their choices are observable (Kim et al., 2021).

Taken together, it can be inferred that socially unfavorable elements, such as unfairness, could be transferred to others, and individuals exhibit sensitivity towards hierarchy, inducing bias in decisions based on both their own and others’ hierarchical positions. However, less is known about unfairness pay-it-forward reciprocity and a question remains regarding how participants adjust their decisions toward unrelated partners with different hierarchies based on their previous experiences of unfairness. Building on prior findings, we hypothesize that individuals are more likely to transmit unfairness to those of lower status than to those of higher status.

To test our hypothesis, we employed a modified dictator game consisting of two rounds with different partners in each round. In the first round, participants acted as recipients receiving either a fair or unfair monetary division from their partners. In the second round, participants acted as dictators and decided how to distribute money to new partners. The two distribution options included a fixed equal payoff and an unfair distribution with random fluctuations from a uniform distribution. During this round, information regarding the partner’s hierarchical status – categorized as high, equal, or low compared to the participant – was displayed on the screen. Remarkably, the hierarchy of both the participants and their partners was predetermined based on their performance in a prior cognitive estimation task (Hays and Goldstein, 2015). The entire experimental procedure was conducted on a metaverse platform, leveraging the metaverse’s potential to enhance laboratory accessibility and facilitate replicability (Gómez-Zará et al., 2023).

The primary objective of this study was to determine how a partner’s hierarchical status influences the unfairness pay-it-forward (PIF) reciprocity in the dictator game within metaverse-based laboratory experiments. Based on prior research, we hypothesized that participants would transmit unfairness to others after experiencing unfair treatment themselves. Moreover, considering that hierarchy could induce decision biases, we expected behavioral changes based on the partner’s hierarchical status relative to the participant: high, equal, or low. Specifically, after receiving an unfair monetary division, participants might be inclined to retaliate against uninvolved individuals, projecting their emotional distress onto them, with this tendency being modulated by the subsequent partners’ hierarchy. If the next partner’s status was higher than that of the participant, we predicted that the participant would distribute more money despite previous unfair treatment, whereas they would allocate less money if the partner’s ranking was lower. Additionally, exploratory analysis was conducted using various personality questionnaires to identify the personality traits influencing individual differences in this phenomenon.

## 2 Materials and methods

### 2.1 Participants

Fifty-three participants were recruited via the Korea University community website for this experiment. Six participants were excluded from the data analysis: Four were excluded due to misunderstanding of the rules or the structure of the experiment, two were excluded because they were suspicious of the cover story. The remaining 47 participants were included in the analyses (23 females, mean age = 23.32 ± 3.5 years). Prior to conducting the study, an *a priori* power analysis was carried out utilizing G*Power (Faul et al., 2007) to determine the appropriate sample size. This analysis was based on the statistical assumptions for a rmANOVA testing for within-factors effects. With an alpha level set at 0.05 and a medium effect size of 0.20, the power analysis recommended a sample size of *N* = 43 to achieve a statistical power of 0.95. This suggested that the sample size employed in this study was sufficient.

Students majoring in psychology, economics, or business administration were screened to prevent any chance of having prior knowledge or familiarity with the task. We ensured that all the participants were naïve to the experimental purpose. Every participant provided written consent and were compensated with KRW 13,000 (approximately equivalent to USD 10). The study was conducted in accordance with all relevant ethical regulations and approved by the Korea University Institutional Review Board.

### 2.2 Experimental procedure

Due to the impact of COVID-19 pandemic, the experiment was conducted remotely using an interactive meta-verse platform, Gather Town.^1^ Although it took place in a digital environment, we carefully designed an online laboratory that closely resembled a traditional, on-site setting. This approach was different from conventional online surveys, which are usually distributed to a large population and completed at the participants’ convenience. Instead, we used a structured method where each participant scheduled a specific time with the experimenter and logged into the web-based laboratory on time. Upon entering the online laboratory through their personalized avatars, participants used their keyboard to navigate their avatar to the experimenter, enabling auditory interaction via a microphone. This real-time communication helped identify and resolve any technical issues related to the screen or button-pressing functionality, as participants accessed the platform using different types of computer devices.

**Figure 1:**
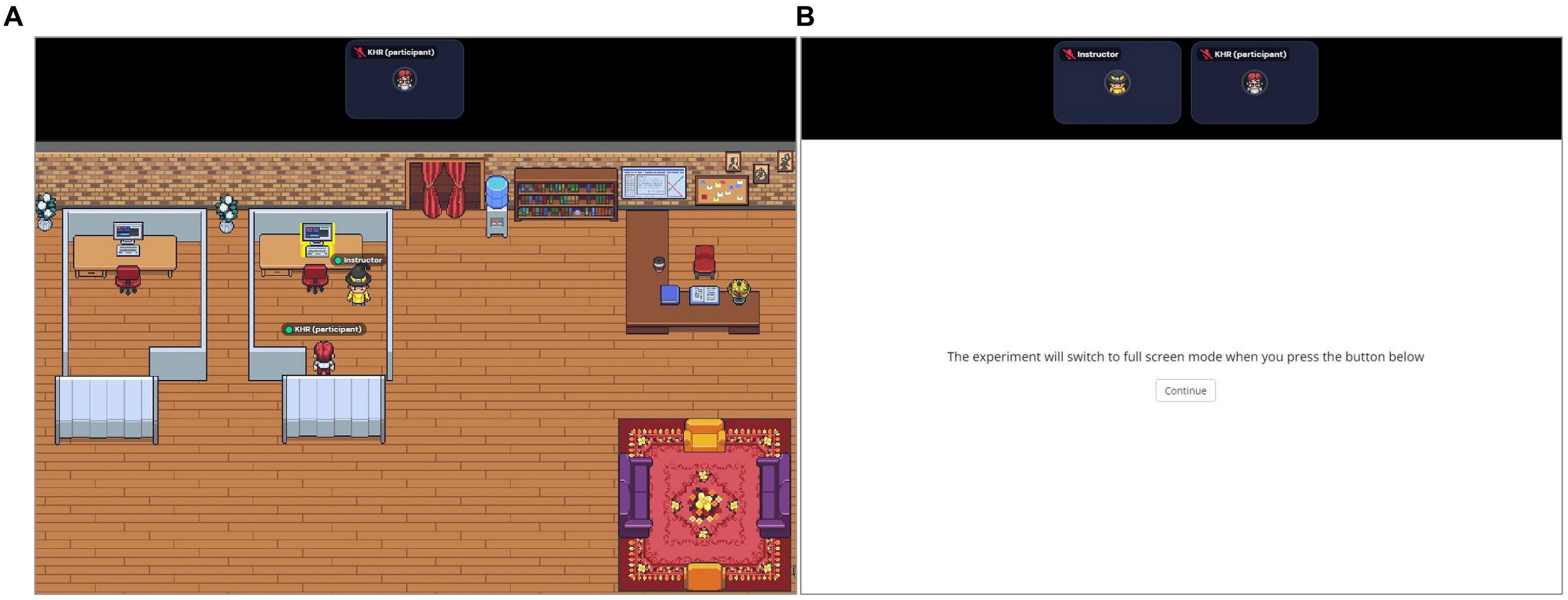
Schematic diagram of metaverse-based experiment. **(A)** Metaverse online laboratory. **(B)** Commencement of the experiment.

Following the experimenter’s guidance, participants were directed to the designated room for the experiment (Figure 1A). These procedures were carefully implemented to replicate the atmosphere and structure of an on-site experiment as closely as possible. Once participants clicked on the “computer” icon, the experiment started in full-screen mode (Figure 1B). Initially, participants were provided with an overall description of the experiment. To ensure consistent understanding among all participants, instructions were delivered in video clips, ensuring uniformity in the explanation. We introduced that the purpose of the study was to investigate the relationship between cognitive performance and cooperativeness within the context of a group teamwork game. Participants were informed that they would engage in three primary activities: (1) cognitive estimation task, (2) modified sequential dictator game, and (3) group teamwork game. They were then asked to complete a series of comprehension questions to verify their understanding of the tasks ahead. Microphone communication was only active during the initial phase before the start of the cognitive estimation task. Once the task began, the microphone was muted, and participants were informed about this change to prevent any potential observational effects. Notably, the group teamwork game, in which participants believed they would take part, did not actually take place. The entire experiment was programmed using jsPsych.^2^

#### 2.2.1 Hierarchy manipulation task

Participants were informed that they would undergo a cognitive task to determine their hierarchy, categorized as high, middle, or low. This task was divided into two sections: the time estimation task and the dot discrimination task. Previous research supports the effective use of simple cognitive tasks to manipulate social hierarchy (Zink et al., 2008; Kim et al., 2021). Participants were told that their performance would establish their rank for a later group teamwork game. However, regardless of their actual performance, every participant was assigned a middle rank. We deliberately designed the task to make self-assessment of performance challenging, aiming to eliminate any suspicion about the hierarchy manipulation procedure.

**Figure 2:**
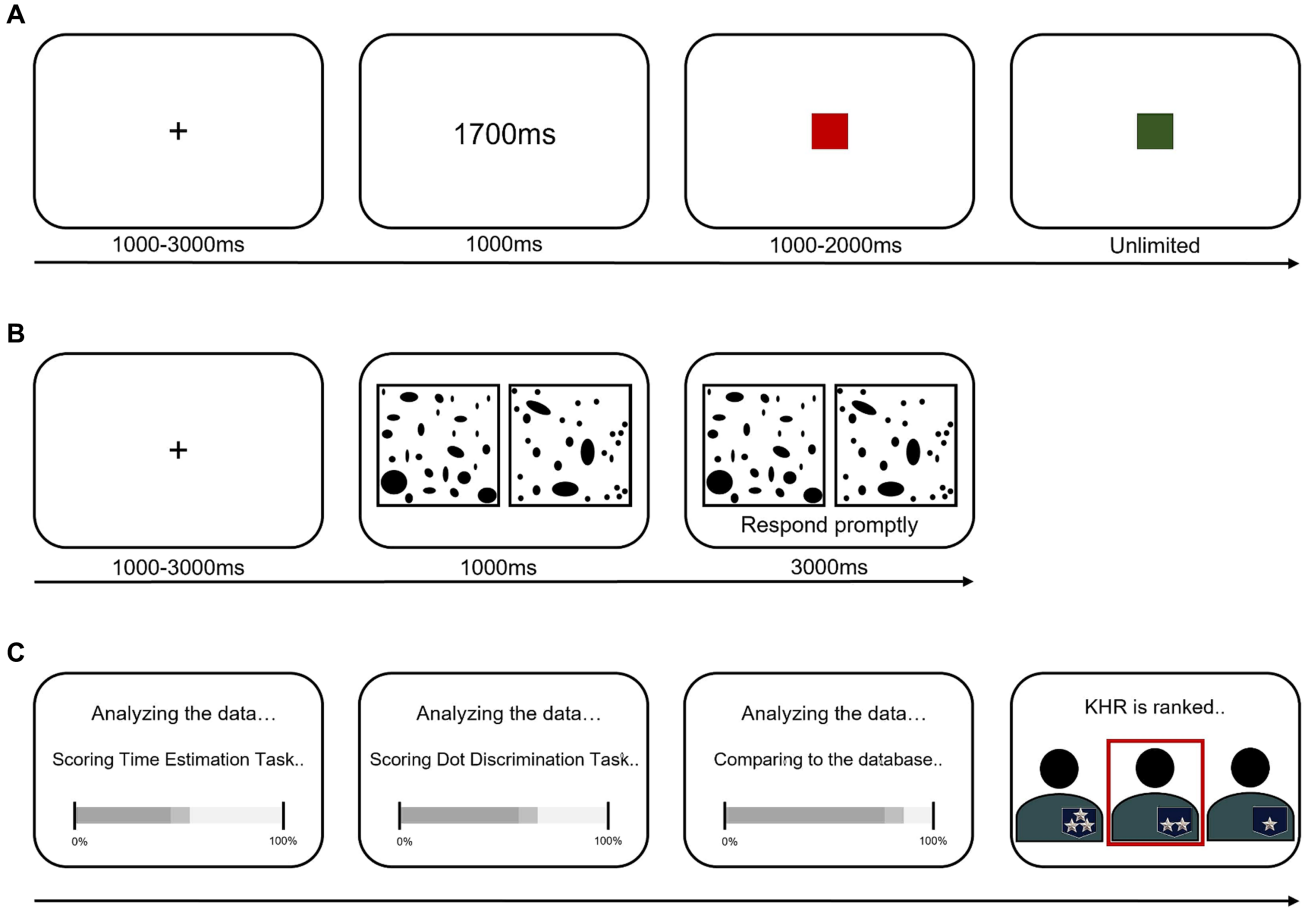
Schematic diagram of hierarchy manipulation task. **(A)** The time estimation task. **(B)** The dot discrimination task. **(C)** Hierarchy assignment depending on the participant’s performance.

The time estimation task assessed how accurately participants could estimate a given time span (Figure 2A). Participants pressed the button as soon as the designated time had passed, indicated by a color change signal (i.e., the square’s color changed from red to green). The target time, varying randomly between 100 ms to 2000 ms, was displayed on the screen at the onset of each trial. A red square then appeared as a readiness signal. When the red square turned green, indicating the start signal, participants estimated the target time before providing their response.

#### 2.2.2 Dot discrimination task

The dot discrimination task evaluated participants’ perceptual abilities to decide which side of a display had more dots, ignoring the size of the dots (Figure 2B). Randomly distributed black dots appeared on a gray background, with 30–60 dots on one side and 3–7 more dots on the other side. Participants selected the side with more dots as quickly as possible. If no response was provided within 1 second, a prompt appeared. If no response was provided within three additional seconds, the trial was marked incorrect, and the next trial began. All participants completed 20 trials for each task.

After these tasks, participants were informed that their hierarchy would be determined comparing their performance to a database of other participants’ performances (Figure 2C). They believed they could be ranked as high, middle, or low rank, represented by the number of stars (i.e., three stars indicating high, two indicating middle, and one indicating low). However, each participant was assigned a middle rank, which was communicated on a screen indicating their calculated hierarchy.

#### 2.2.3 Modified sequential dictator game

Following the cognitive estimation task and the hierarchy assignment, participants were informed that their ranking would only be used in the group teamwork game and not in the dictator game. All participants, represented by avatars in rank-appropriate uniforms, participated in a modified sequential dictator game. This cover story was provided because the study aimed to investigate how perceived hierarchy might regulate the effect of unfairness on subsequent decisions (i.e., unfairness PIF reciprocity). That is, such approach was taken to explore how subtle hierarchy might influence decision-making, particularly when unfairness is experienced.

**Figure 3:**
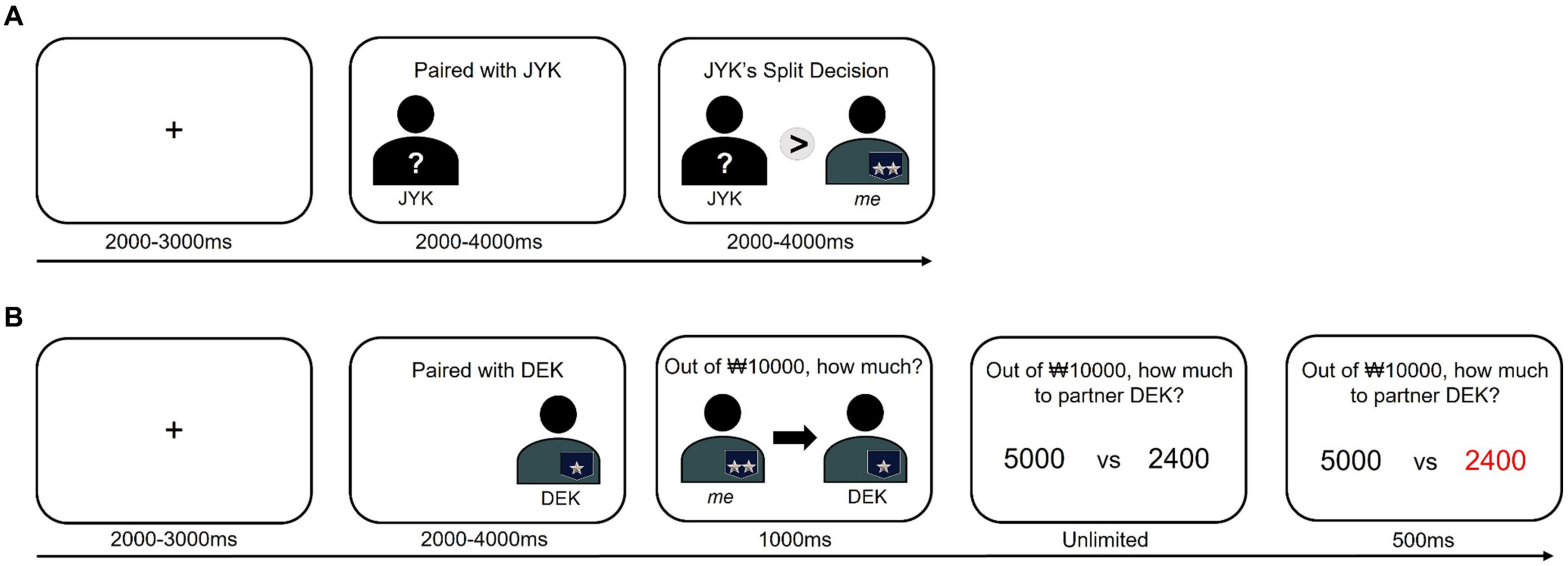
Modified sequential dictator game where each trial comprises two rounds. **(A)** In the first round, the participant (e.g., “me” on the right side) performed as a recipient, viewing the distribution result made by the dictator (e.g., “JYK” on the left side). **(B)** In the second round, the participant (e.g., “me” on the left side) performed as a dictator, deciding how much money to allocate to the partner (e.g., “DEK” on the right side) out of $10.

In the present study, we used a modified sequential dictator game, where each trial consisted of two dictator games. Participants took part as recipients in the first dictator game (DG1) and as dictators in the second dictator game (DG2) (Figure 3). Prior to the sequential dictator game, participants drew for which role to play in each game (e.g., dictator in DG1 and recipient in DG2, or vice versa), believing that the roles they would play in each game were randomly determined. However, it was predetermined in advance that every participant would act as a recipient in DG1 and as a dictator in DG2.

Prior to conducting the main study, a preliminary pilot study was carried out with 24 participants to refine the factors for the main experiment. In the pilot study, a significant interaction effect was observed between the fairness of offers in DG1 and the hierarchy of partner in DG2 ($F_{\left(1,23\right)}$ = 11.606, *p* < 0.01, partial $\eta^{2}$= 0.335). However, in the pilot design, the DG2 partner’s hierarchy included only equal and low conditions (relative to the participant), with the absence of a high condition. This limitation hindered a comprehensive understanding of how unfairness is transmitted across different hierarchical levels. Hence, we added a higher status condition to DG2 in the main experiment. Additionally, the broad range of the unfair options (2.4% ~ 48% of the endowment) in the pilot study also posed a limitation for interpretation. To address this, we narrowed the range of unfair proposals (2.4% ~ 24% of the endowment) to distinguish them more clearly from fair proposals.

Therefore, the main experiment employed a 2 × 3 within-participant factorial design, with the first factor referring to the DG1 fairness (fair vs. unfair), and the second factor referring to the DG2 partner’s hierarchy (high vs. equal vs. low). High, Equal, and Low indicate the partners’ superior, equivalent, and inferior status relative to the participant, respectively. Each condition (FH, FE, FL, UH, UE, UL) consisted of 20 trials (120 trials in total).

In DG1 (Figure 3A), following the display of a jittered black fixation cross on a gray background (duration 2–3 s, uniformly distributed), the information of the DG1 partner (i.e., dictator) was presented on the left side (duration 2–4 s). Next, the dictator’s distribution decision was shown in the center using mathematical symbols (i.e., “=” or “>”; duration 2–4 s) between the partner’s avatar on the left and the participant’s avatar on the right side of the screen. Participants received either fair (i.e., “=,” indicating a 50–50 split of the resources) or unfair (i.e., “>,” indicating the partner has taken more than a 50% share) offers. Here, the dictator’s hierarchical status was concealed with a question mark to avoid additional complexity related to the dictator’s status, ensuring that hierarchy was inferred solely from the game rules: only the dictator has the authority to allocate resources, while the recipient cannot object.

In DG2 (Figure 3B), the introduction of a new round was signaled by a jittered black fixation cross on a gray background (duration 2–3 s). Next, the information of the DG2 partner (i.e., recipient) was displayed on the right side of the screen (duration 2–4 s). Then, the question asking how much out of KRW 10,000 the participant would split appeared at the top of the screen (duration 1 s). Two distribution options were provided: one being an equal split (50–50), and the other being an unequal distribution favoring the participant. That is, the fair option was fixed at KRW 5,000, while the unfair option varied from KRW 240 to KRW 2,400 (10 unfair offers: KRW 240, 480, 720, 960, 1,200, 1,440, 1,680, 1920, 2,160, 2,400).

To prevent participants from recognizing that the offers in DG1 were predetermined, they were informed that the decisions were made by previous participants (Boksem and De Cremer, 2010; Wu et al., 2015) and that their responses in DG2 would be provided to future participants as well. Additionally, to ensure sincere responses, participants were informed that one trial from DG1 and one trial from DG2 would be randomly selected, and the average amount would be given as an additional incentive. However, in DG1, participants only saw symbols (‘=’ or ‘>’) indicating whether they receive an equal share (5,000 KRW) or less, without knowing the exact amount. Therefore, we provided a fixed total payment of 13,000 KRW to all participants.

#### 2.2.4 Group teamwork game

Although the group teamwork game did not actually occur, we included this task to enhance the plausibility of the cover story. Prior to the dictator game, participants were informed that the hierarchy determined in the cognitive estimation task would be applied in the group teamwork game and that they would gather with some partners from the dictator game as a team to undertake the task. No specific information about the group teamwork game was provided to prevent participants from having unnecessary expectations.

After completing the modified sequential dictator game, participants were instructed to fill out questionnaires measuring individual differences in personality and were asked to answer a few questions in the interview. Comprehensive questions were included to ensure that there was no misunderstanding of rules or structure. Specifically, participants were asked to write down what they thought the study was about and its purpose. Consequently, four participants who conducted the experiment without understanding the structures and two participants who noticed that the study aimed to investigate decision-making under unfair treatment and that the hierarchy was manipulated to a middle rank regardless of actual performance were excluded from the analysis.

All participants were manipulated to be assigned a middle rank, and in the sequential dictator game, they were assigned the role of recipient in the first round and dictator in the second round. This manipulation ensured that all participants experienced the same conditions (fair or unfair prior interactions and partners of higher, equal, and lower ranks) and the same number of trials, which were necessary for unbiased statistical analysis based on balanced trial numbers in studies with limited situations and sample sizes, such as fMRI experiments. Given the deception included in this study, debriefing sessions were conducted after the experiment, during which participants were informed of the deception, and consent forms detailing the deception were obtained. They were also debriefed about deception regarding the absence of the group teamwork game. Consequently, participants understood the purpose of the study, and those with additional questions were encouraged to contact us for further information. This procedure was approved by the Korea University Institutional Review Board (IRB).

### 2.3 Post-experiment questionnaires

The post-experiment questionnaires included items from the Personal Sense of Power (Anderson et al., 2012), Self-Esteem Inventory (Coopersmith, 1967), Toronto Empathy Questionnaire (Spreng et al., 2009), and Fear of Negative Evaluation (Leary, 1983) scales. The Personal Sense of Power assesses one’s ability to influence another person or others (Anderson et al., 2012) and consists of eight items, such as “My ideas and opinions are often ignored” (reverse-scored), with a 7-point Likert scale (1 = strongly disagree, 7 = strongly agree). The Self-Esteem Inventory, measuring attitudes toward the self in various areas (Coopersmith, 1967), was composed of 25 items divided into four categories: personal relation, leadership and popularity, self-enhancement, and assertiveness and confidence (Yeun and Kwon, 2007). In particular, self-enhancement consists of questions such as “There are lots of things about myself I’d change if I could” (reverse scored). The participants responded on a 4-point Likert scale (1 = strongly disagree, 4 = strongly agree). The Toronto Empathy Questionnaire was used to measure one’s empathy level, composed of 16 items such as “It upsets me to see someone being treated disrespectfully” with a 5-point Likert scale (0 = strongly disagree, 4 = strongly agree). The Fear of Negative Evaluation scale measures individual’s apprehensiveness of negative evaluation by other people, including 12 items like “I am afraid that others will not approve of me” with 5-point Likert scale (1 = strongly disagree, 5 = strongly agree). Exploratory Pearson correlation analyses were conducted to investigate the relationships between personality traits measured using the questionnaires and behavioral indices from the dictator game.

### 2.4 Behavioral data analyses

As the participants were allowed to respond with an unlimited amount of time in the decision phase, we first checked the response time and screened out unusually slow trials as outliers using the median absolute deviation methods (Leys et al., 2013). Separately, for each of the conditions, we calculated the average chosen amount across the trials in which the participant decided to split the partner. We then normalized the average chosen amount across conditions within each subject for between-subject comparisons. Two participants were excluded from the data analysis using the normalized average chosen amount because they chose fair options in every trial; therefore, their normalized average chosen amount could not be calculated. Then, to investigate the overall effect of DG1 fairness and DG2 partner’s hierarchy, we entered the condition-wise normalized average chosen amount as dependent variables in a repeated-measures ANOVA (rmANOVA) with fairness (Fair, Unfair) and DG2 partner’s hierarchy (High, Middle, Low) as within-subject factors. Moreover, we conducted a paired-samples *t*-test to examine the simple main effects that potentially contribute to the statistically significant interaction effects.

We also examined the individual differences in the effect of DG1 fairness, DG2 partner’s hierarchy, their interactions, and the chosen amount of unfair option in each trial by employing a generalized linear model (GLM), using the “glmfit” function in MATLAB. Prior to performing linear regression, DG1 fairness was coded as either 1 (fair) or − 1 (unfair), and the DG2 partner’s hierarchy was coded as either 1 (high), 0 (equal), or − 1 (low). Then, DG1 fairness, DG2 partner’s hierarchy, and the interactions of the two were fitted to the chosen amount to yield participant-specific indices of fairness sensitivity ($\beta_{\text{Fairness}}$), hierarchy sensitivity ($\beta_{\text{Hierarchy}}$), interaction sensitivity ($\beta_{\text{Fairness}*\text{Hierarchy}}$), and chosen amount of unfair option sensitivity ($\beta_{\text{unfair}\;\text{option}}$). The parameters generated from the linear regression were correlated with personality traits in correlation analyses to investigate any associations between them.

Behavioral analyses were performed using MATLAB (version R2021a), IBM SPSS Statistics (version 26), R Studio (version 4.1.0), and Python (version 3.10).

## 3 Results

### 3.1 Monetary distribution

**Figure 4:**
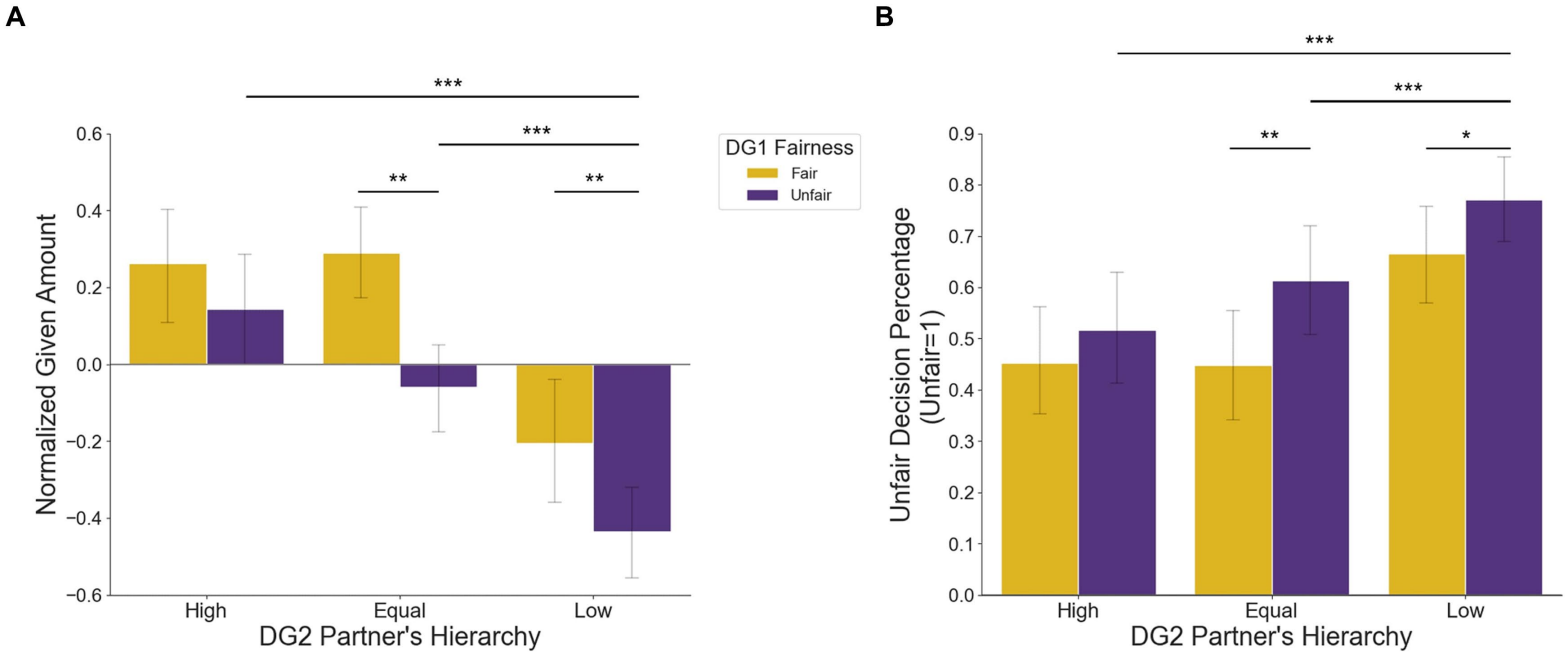
Behavioral results. **(A)** 2 (DG1 fairness: Fair or Unfair) × 3 (DG2 partner’s hierarchy: High vs. Equal vs. Low) rmANOVA on the normalized chosen amount and **(B)** on the average percentage of choosing unfair option.

To examine the effect of DG1 fairness and the DG2 partner’s hierarchy on the distribution decision, we performed a 2 (DG1 fairness: fair or unfair) × 3 (DG2 partner’s hierarchy: high, equal, or low) repeated-measures ANOVA (rmANOVA) on the normalized chosen amount (Table 1). A significant main effect of DG1 fairness was found ($F_{\left(1,44\right)}$ = 14.166, *p* < 0.001, partial $\eta^{2}$= 0.244), indicating the participants’ tendency to be fairer when the previous partner had offered fair treatment, while being unfair when treated unfairly by the preceding partner. The main effect of the DG2 partner’s hierarchy was also significant ($F_{\left(1,44\right)}$ = 13.208, *p* < 0.001, partial $\eta^{2}$= 0.381), and the behavioral patterns suggested that the participants were more likely to distribute more money to those with higher ranking, whereas less to those with lower ranking. The interaction between DG1 fairness and the DG2 partner’s hierarchy was also significant ($F_{\left(1,44\right)}$ = 5.584, *p* = 0.007, partial $\eta^{2}$= 0.206), indicating that the influence of DG1 fairness on the decision differed depending on which ranking the DG2 partner possessed (Figure 4A). To determine the cause of the interaction, we performed a 2 × 2 rm. ANOVA with DG1 fairness and DG2 partner’s hierarchy as factors for each pair of DG2 partners’ hierarchy (i.e., High-Equal, High-Low, Equal-Low). The results showed that the DG1 fairness × DG2 partner’s hierarchy was significant for High and Equal ($F_{\left(1,44\right)}$ = 9.342, *p* = 0.004, partial $\eta^{2}$ = 0.175) and High and Low ($F_{\left(1,44\right)}$ = 6.038, *p* = 0.018, partial $\eta^{2}$ = 0.121), but not for Equal and Low ($F_{\left(1,44\right)}$ = 2.665, *p* = 0.110, partial $\eta^{2}$ = 0.057).

**Table 1:** rmANOVA results: the upper table indicates the result for Figure 4A (normalized given amount) and the lower table indicates result for Figure 4B (unfair decision percentage).

Source	*F*	Sig.	Partial Eta Squared
*Main effect*			
DG1 fairness	14.166	***p*** **< 0.001**	0.244
DG2 partner’s hierarchy	13.208	***p*** **< 0.001**	0.381
*Interaction effects*			
DG1 fairness * DG2 partner hierarchy	5.584	***p*** = **0.007**	0.206

Source	*F*	Sig.	Partial Eta Squared
*Main effect*			
DG1 fairness	13.791	***p*** = **0.001**	0.231
DG2 partner’s hierarchy	11.979	***p*** **< 0.001**	0.347
*Interaction effects*			
DG1 fairness * DG2 partner hierarchy	4.722	***p*** = **0.014**	0.173

We also performed the same rmAVOVA analyses on the average percentage of participants choosing an unfair option to ensure that the same result would be yielded. As expected, significant main effects of both DG1 fairness ($F_{\left(1,44\right)}$ = 13.791, *p* = 0.001, partial $\eta^{2}$ = 0.231) and DG2 partner’s hierarchy ($F_{\left(1,44\right)}$ = 11.979, *p* < 0.001, partial $\eta^{2}$ = 0.347) as well as a significant interaction effect ($F_{\left(1,44\right)}$ = 4.722, *p* = 0.014, partial $\eta^{2}$ = 0.173) were found (Figure 4B). Moreover, we performed the same rmANOVA analyses for the response time to check if there was any difference in response time depending on the condition, but no significant effect on the RT data was revealed.

*Post-hoc* paired-sample *t*-tests (Figure 4A) showed no significant difference between Fair-High (*M* = 0.262, *SD* = 0.499) and Unfair-High (*M* = 0.144, *SD* = 0.515) conditions, [$t_{\left(44\right)}$ = 1.930, *p* = 0.060, C.I. = (−0.005, 0.242)]. This implies that the money distributed to those with high rankings was not strongly affected by the DG1 fairness in the previous round. However, the participants showed significant difference between Fair-Equal (*M* = 0.290, *SD* = 0.401) and Unfair-Equal (*M* = −0.059, *SD* = 0.402) conditions, [$t_{\left(44\right)}$ = 3.912, *p* < 0.001, C.I. = (0.169, 0.529)], as well as between Fair-Low (*M* = −0.204, *SD* = 0.549) and Unfair-Low (*M* = −0.435, *SD* = 0.432) conditions, [$t_{\left(44\right)}$ = 3.605, *p* = 0.001, C.I. = (0.102, 0.360)]. This suggests that when the DG2 partner’s hierarchy is equivalent or inferior, DG1 fairness matters in decision making. Considering our interest in how unfairness could spread differently with regard to the partner’s hierarchy, paired-sample t-tests for unfair conditions were conducted. Significant difference between Unfair-High (*M* = 0.144, *SD* = 0.515) and Unfair-Low (*M* = −0.435, *SD* = 0.432) conditions was shown, [$t_{\left(44\right)}$ = 4.676, *p* < 0.001, C.I. = (0.330, 0.829)], as well as between Unfair-Equal (*M* = −0.059, *SD* = 0.402) and Unfair-Low (*M* = −0.435, *SD* = 0.432) conditions, [$t_{\left(44\right)}$ = 4.578, *p* < 0.001, C.I. = (0.210, 0.541)]. However, there was a marginally significant difference between Unfair-High (*M* = 0.144, *SD* = 0.515) and Unfair-Equal (*M* = −0.059, *SD* = 0.402), [$t_{\left(44\right)}$ = 2.010, *p* = 0.051, C.I. = (−0.001, 0.407)]. This indicates that unfairness is more likely to spread toward those with a lower ranking compared to the other rankings.

### 3.2 Correlation analysis

**Figure 5:**
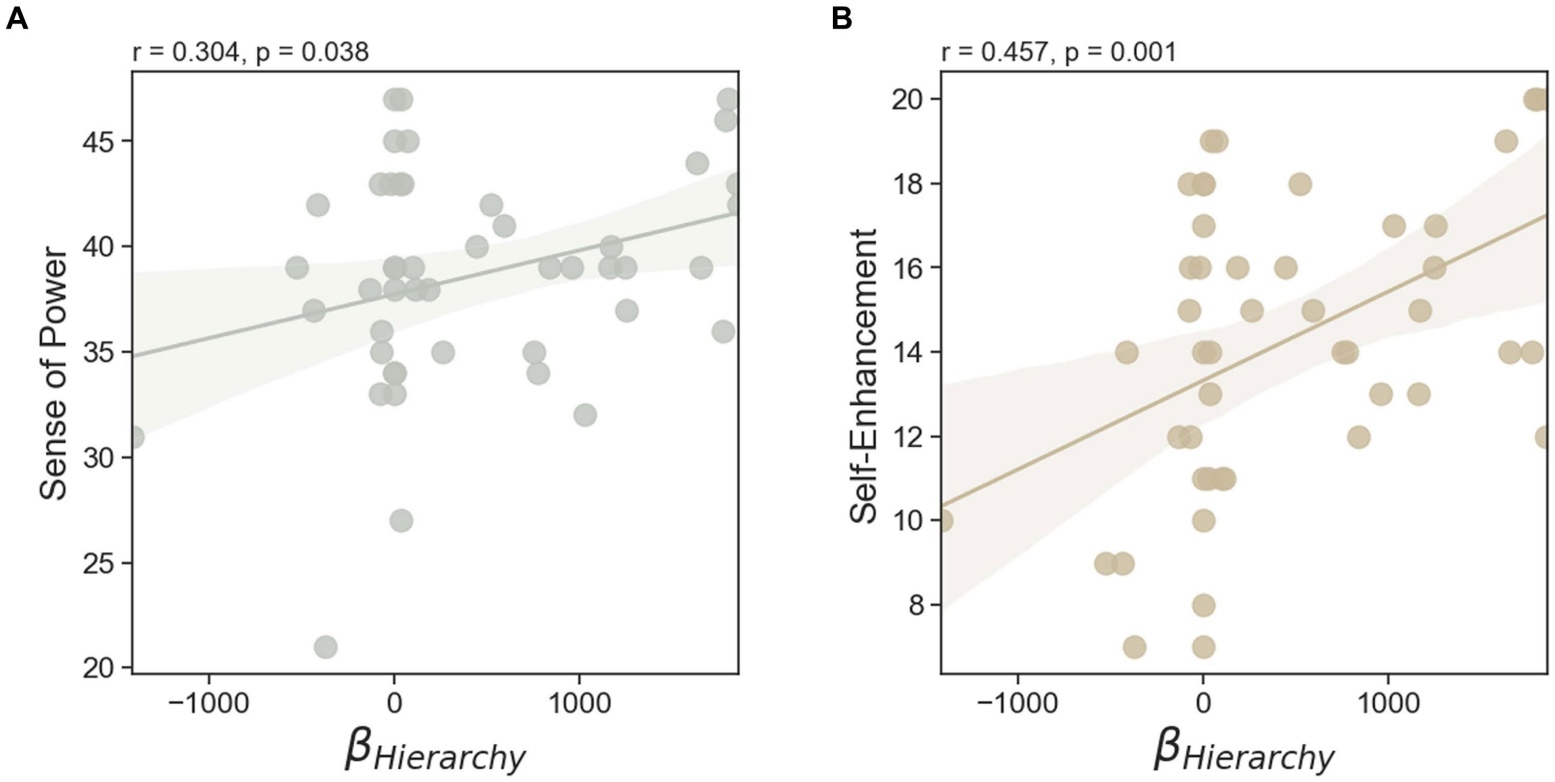
Individual differences in personality traits predicting one’s sensitivity to the partner’s hierarchy. **(A)** People with higher sensitivity to the partner’s hierarchy showed higher personal sense of power (SoP) score and **(B)** higher self-enhancement (SEM) score. All results are based on two-tailed Pearson correlation analyses and the line shadow indicates the 95% confidence interval.

**Figure 6:**
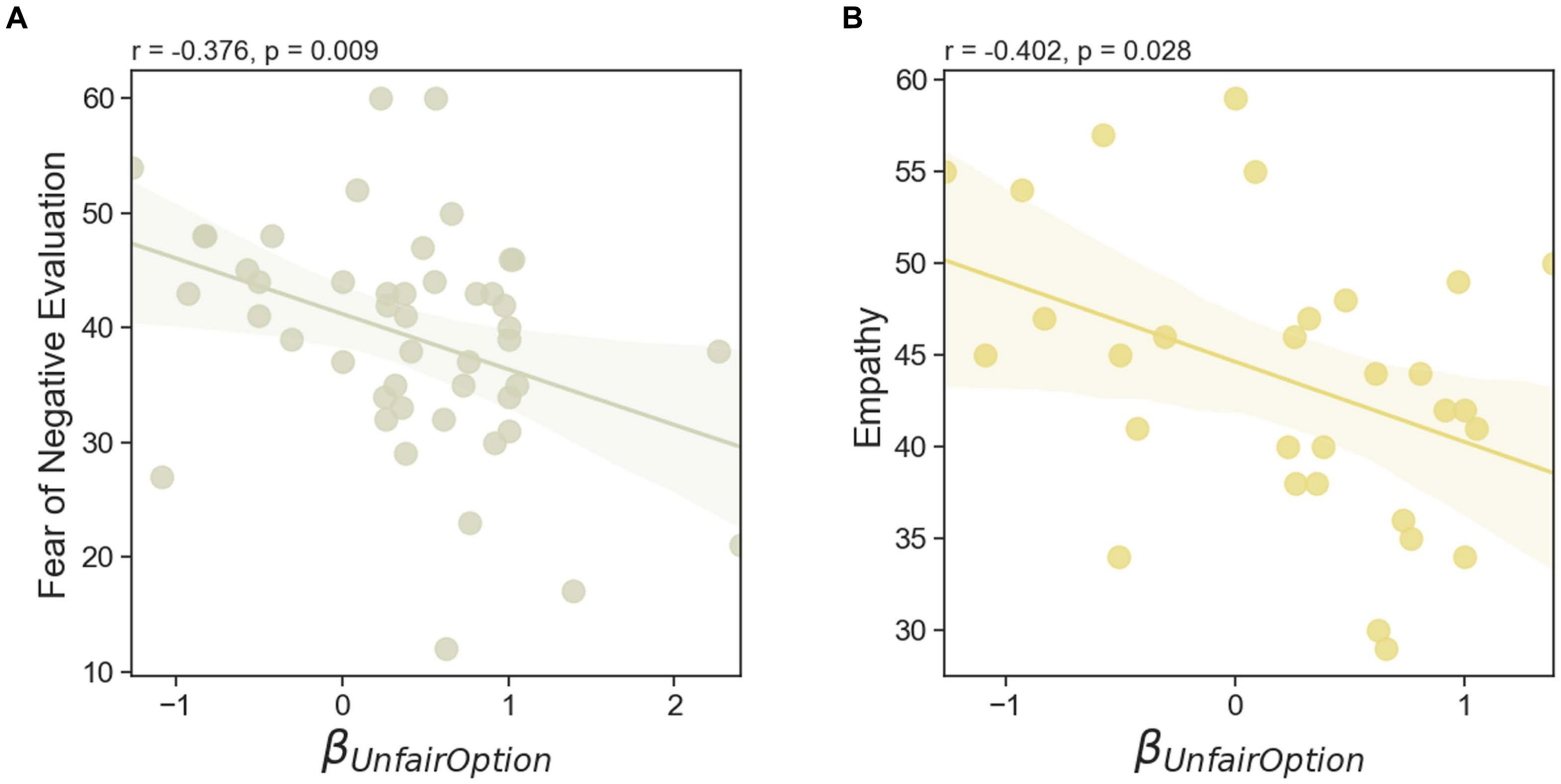
Individual differences in personality traits predicting the degree to which participants are sensitive to the unfairness of the allocation offered. Unfair option parameter indicates increased tendency of splitting fair option to the DG2 partner as the unfair option increases. Those with higher unfair option sensitivity were more likely to split more money to the partner when the amount of unfair option decreased. **(A)** Participants with high degree of such parameters were negatively correlated with empathy and **(B)** fear of negative evaluation (FNE). Both results are based on two-tailed Pearson correlation analyses and the shadowed line indicates the 95% confidence interval.

We examined whether there were any relationships between individual differences in DG1 fairness sensitivity, DG2 partner’s hierarchy sensitivity, and unfair option amount sensitivity from the task data and personality traits such as sense of power (SoP), self-enhancement (SEM), fear of negative evaluation (FNE), and empathy scores. A significant positive correlation was observed between sensitivity to the DG2 partner’s hierarchy and SoP (Pearson’s $r_{\left(46\right)}$= 0.304, *p* = 0.038, two-sided) (Figure 5A). In addition, an individual’s sensitivity to hierarchy was positively correlated with SEM (Pearson’s $r_{\left(46\right)}$= 0.457, *p* = 0.001, two-sided) (Figure 5B). By contrast, unfair option amount sensitivity (i.e., tendency to offer a fair amount to the partner when the unfair option amount increases) was negatively correlated with FNE (Pearson’s $r_{\left(46\right)}$= −0.376, *p* = 0.009, two-sided) (Figure 6A) and empathy (Pearson’s $r_{\left(29\right)}$= −0.402, *p* = 0.028, two-sided) (Figure 6B).

## 4 Discussion

The present study aimed to examine whether and how individuals spread unfairness to innocent third parties when the hierarchical status of their partners is visible. Specifically, we investigated the impact of unjust treatment from prior interactions on subsequent interactions with new partners of varying rankings. Based on previous findings and theoretical frameworks, we hypothesized that individuals who experienced inequitable resource distribution from previous partners would forward unjustness more heavily on those of lower ranking compared to those of higher ranking. To test this hypothesis, we employed a modified sequential dictator game. Each participant performed two games: one as a recipient and the other as a dictator. In the first round, participants, as recipients, observed whether their partner’s distribution decision was fair (i.e., equal amount for both) or unfair (i.e., less amount to the participant). In the second round, paired with a new partner, participants decided whether to distribute money fairly (i.e., equal amount to the partner) or unfairly (i.e., less amount to the partner), based solely on the partner’s status.

Consistent with our prediction, participants who experienced a disadvantageous distribution in the first round (DG1) were more likely to allocate fewer resources to partners of lower rankings in the second round (DG2). The results demonstrated that people transmit both socially desirable and undesirable behaviors. Specifically, when the partner’s ranking was equivalent to that of the participant, participants tended to mirror the preceding partner’s choices, resulting in fair treatment begetting fair treatment and unfair treatment begetting unfair treatment. This finding supports the concept of “pay-it forward reciprocity” or “indirect reciprocity,” as supported by numerous previous studies (Gray et al., 2014; Hu et al., 2015; Wu et al., 2015; Strang et al., 2016; Hu et al., 2018). Furthermore, the successful replication of pay-it-forward reciprocity in our study, particularly within an online context, suggests that experiments conducted in a metaverse can effectively create an environment similar to traditional on-site experiments.

Our study extends the existing literature by demonstrating that individuals are not only sensitive to the unfairness they experience in prior interactions but also adjust their behavior in subsequent interactions based on the hierarchical status of their partners. This behavioral inclination, described as “you pinch me then I will pinch someone else,” was conditional upon the status of the partner in the second round. In other words, although provoked by unequal distribution, the urge to reciprocate was modulated by the partner’s hierarchy: participants were more likely to pass on the unfairness to lower-ranked individuals than to higher-ranked ones. In addition, the motivation to express hierarchy-biased decisions was associated with SoP and SEM, while sensitivity to unfair options was related to FNE and empathy. Considered together, these findings provide empirical evidence for the framework of unfairness PIF reciprocity in hierarchical contexts, where individuals’ responses to received patterns of behavior are influenced by their partner’s hierarchical status.

Experiencing unfairness can trigger perceptions of injustice and affect one’s attitude or behavior, ultimately biasing subsequent decision-making (Lind and Tyler, 1988; Van den Bos, 2005, 2018). When treated unfairly, people tend to treat others unfairly in return, following the strategy of “an eye for an eye, a tooth for a tooth” (Wu et al., 2015). However, this tendency extends beyond direct revenge, affecting even uninvolved individuals in anonymous interactions. Previous studies have shown that perceptions of unfairness lead to negative outcomes, such as anger, depression, and violent behavior (Klandermans, 1997; Meier et al., 2009). Being treated unfairly by group members signals that one is not valued as an individual nor as a member of a group or a society which one belongs (Lind and Tyler, 1988; Van den Bos, 2018). Since humans inherently strive for social connection, unfair treatment that implies social exclusion is particularly painful (Eisenberger et al., 2003). The sense of fairness seems to be deeply rooted in human nature, as evidenced by standards that emerge early in infancy (Schmidt and Sommerville, 2011; Sloane et al., 2012; Buyukozer Dawkins et al., 2019), which forms the foundation of moral cognition. Hence, as human beings, we expect others to be reciprocally fair. This expectation explains why fair-dependent effects on behavior appear only when partners are perceived as real people, not as computers (Hu et al., 2018). When the expectation of mutual fairness is unmet and a prediction error occurs, it activates an internal alarm system, prompting individuals to evaluate their surroundings and make sense of provoking events (Van den Bos, 2018). The gap between expected and actual fair treatment increases the activation of alarm systems, leading to negative emotional responses such as anger, anxiety and depression (Mikula et al., 1998; Urbanska et al., 2019). To resolve this imbalance and alleviate heightened alarm responses, individuals may redirect their emotional burden from prior partners to uninvolved others.

Previous research has shown how hierarchy influences perceptions of unfairness using the Ultimatum Game - individuals who achieved high rankings through cognitive tasks are more likely to reject unfair offers from their partners (Hu et al., 2015), and perceptions of unfairness vary based on their own socioeconomic status (Zheng et al., 2017). However, the impact of a recipient’s hierarchy, even when manipulated within the experiment, on PIF reciprocity has not been clearly investigated. Extending the prior findings, our study reveals that individuals are highly responsive to either socially favorable or unfavorable behaviors toward strangers based on preceding events, and that one’s motivation to act unfairly is adjusted according to the partner’s hierarchy in subsequent encounters.

Moreover, considering the effect of hierarchy on PIF reciprocity, we explored how individual differences in SoP and SEM relate to sensitivity to hierarchy ($\beta_{\text{Hierarchy}}$). Our results indicate that the tendency to distribute more resources to high-ranked partners and fewer resources to low-ranked partners, regardless of previous treatment, is more evident among individuals with higher levels of SoP and SEM. Both SoP and SEM are linked to power-oriented tendencies and narcissism (Farwell and Wohlwend-Lloyd, 1998; Miller and Campbell, 2011; Nevicka and Sedikides, 2021). SoP refers to the perception of one’s ability to influence other people, which varies across individuals even when they have control over the same resources or socio-structural positions (Bandura, 1999; Anderson et al., 2012). SEM reflects an inclination toward inflated and positively biased self-views, often deviating from reality in ways that are improbable or logically impossible (Leary, 2007; Zell et al., 2023). Even when external indicators describe their ability or attributes, people with elevated SEM evaluate these indicators more favorably than they are supposed to Dufner et al., (2019). This suggests that, despite being assigned a middle-rank, those with high SEM perceive themselves as having a higher rank due to their tendency to overestimate their abilities. This self-enhancement leads to hierarchy-biased decision-making. Consequently, under the influence of the DG2 partner’s hierarchy, the fairness results from DG1 were not salient enough to impact their distribution decisions.

Another interesting finding of this study is that the participants adjusted their decisions based on the varying amounts of the unfair option across trials (i.e., KRW 240–2,400). This tendency was measured by the chosen amount of unfair option sensitivity ($\beta_{\text{unfair}\;\text{option}}$), where a higher value indicates a greater inclination to distribute more resources (i.e., be fairer) as unfair option amounts increase. Participants with a high degree of this parameter chose an unfair split when the disparity between the fair and unfair options were large, maximizing their own profit. Conversely, they were more likely to distribute equal amounts when the difference between options decreased, reducing potential benefits from choosing the unfair option. Furthermore, individual differences in FNE and empathy were negatively correlated with this tendency. This result suggest that participants inclined toward unfair options when disparities were large were less sensitive to inequity and exhibited lower levels of FNE and empathy. FNE arises from apprehension about being judged negatively by others (Carleton et al., 2006). While most people occasionally worry about how they would be viewed by others, individuals differ in the degree to which they adjust their behaviors in accordance with others’ judgments. Those highly concerned about negative evaluations tend to act in ways that prevent unfavorable impression (Leary, 1983; Carleton et al., 2006). To avoid negative public images, they employ strategies such as prosocial behavior, self-presentation, conformity, and social facilitation (Schlenker, 1980). Previous research has shown that empathy positively predicts prosocial behavior, including fairness to others (Bieleke et al., 2017). Empathetic individuals are more responsive to unfairness directed at others, motivating them to act based on the victim’s feelings (Urbanska et al., 2019). For instance, empathy has been shown to relate positively to fairness in groups of nursing students (Woo and Park, 2019) and teachers (Hong et al., 2022).

In light of this empirical evidence and theoretical insights, we can infer that individuals who are highly concerned about being perceived negatively may distribute equal amounts of resources to their partners when the unfair option is particularly unfavorable. This behavior is likely to avoid being reflected as selfish individuals who maximize their own profit at the expense of their partners. The underlying intention can be interpreted as a preference for maintaining a favorable public image (e.g., fair person image) over monetary benefit (e.g., one’s possible benefit by splitting less to the partner), especially among those with high levels of FNE. Conversely, those with low FNE place less emphasis on this public image. In addition, empathy appears to motivate individuals to act fairly by enabling them to perceive partners’ negative feelings when given unequal amounts. Highly empathetic individuals, absorbing others’ feelings and blur the boundary between self and other, may experience distress and perceive unfairness stemming from their own unfair decisions. Thus, individuals with high empathy are more likely to avoid placing their partners in a disadvantageous position and opt to distribute resources equally when the alternative is intensely unfair.

Taken together, the current study suggests that people spread unfairness to uninvolved, anonymous others, especially those with lower hierarchical status. Sensitivity to the partner’s hierarchy is particularly noticeable among those with higher levels of SoP as well as among those with higher SEM. Moreover, sensitivity to degree of unfairness varied among participants, being more prominent among those with lower empathy and among those with lower FNE. Confirming our *a priori* prediction, individuals tend to treat others in a way that they were treated previously: when treated unfairly, they transmit this unfairness to others, modulated by the counterpart’s hierarchical status. We believe that this study provides empirical evidence of unfairness PIF reciprocity in hierarchical contexts. Moreover, a future neuroimaging study could further explore the neural mechanisms that underpin how people perceive and incorporate varied social information about unfairness and hierarchy to arrive at attuned decision-making.

A few limitations should be considered further for the future study. First, the cultural and national context in which this study was conducted may influence the generalizability of the findings. While the phenomenon of the transmission of both socially desirable and undesirable behaviors has been observed globally, this experiment was conducted exclusively with Korean participants. The collectivist culture of East Asia may have impacts on the results, which might differ in individualistic cultures, such as those prevalent in many Western countries. It would be important for future research to explore whether similar patterns of behavior are observed in participants from different cultural backgrounds, considering the potential influence of cultural dimensions on experimental results. Second, the age of the participants is another factor to consider. This study was conducted with university students in their twenties. It is important to investigate whether the impact of perceived unfairness and hierarchical status on decision-making varies across different age groups. The reactions to unfair treatment and subsequent behaviors may differ significantly between younger and older adults, influenced by varying life experiences and developmental stages. Lastly, the online nature of this study due to the COVID-19 pandemic introduces another limitation. Although the results aligned with previous research, confirming the validity of online experiments, it is crucial to examine whether these findings hold in face-to-face interactions. The dynamics of in-person interactions could reveal additional nuances in unfairness PIF in a hierarchical context, which may not be fully captured in an online experiment. Future studies should consider replicating this research in physical settings to validate and extend these findings.

Propagation of unfairness is a phenomenon observed globally, particularly in hierarchically structured medical environments like nursing organizations. This phenomenon manifests as a longstanding chain of behaviors. The significance of this study lies in providing insights into developing policies and programs aimed at preventing the downward propagation of unfairness within hierarchy-based organizational structures. By examining the psychological mechanisms underlying such propagation, this research paves the way for enhancing the overall mental health and well-being of individuals across various professions who are impacted by this enduring pattern of unfairness. Therefore, this study holds importance at the intersection of social, health, and occupational psychology, providing a comprehensive understanding of the unfairness PIF. This understanding is crucial for developing strategies that not only address the phenomenon itself but also offer policy insights to break this vicious circle, ultimately promoting a cooperative and more equitable professional environment.

## Data availability statement

The raw data supporting the conclusions of this article will be made available by the authors, without undue reservation.

## Ethics statement

The studies involving humans were approved by Institutional Review Board of Korea University. The studies were conducted in accordance with the local legislation and institutional requirements. The participants provided their written informed consent to participate in this study.

## Author contributions

HyK: Conceptualization, Formal analysis, Investigation, Methodology, Visualization, Writing – original draft. JK: Conceptualization, Formal analysis, Methodology, Writing – original draft. DK: Conceptualization, Methodology, Writing – original draft. HaK: Conceptualization, Funding acquisition, Methodology, Resources, Supervision, Writing – original draft, Writing – review & editing.

## Footnotes

^1^ https://www.gather.town

^2^ https://www.jspsych.org
